# Methyl­ammonium tetra­fluoridoborate 18-crown-6 clathrate

**DOI:** 10.1107/S1600536811054432

**Published:** 2011-12-23

**Authors:** Yu Jin

**Affiliations:** a Ordered Matter Science Research Center, Southeast University, Nanjing 211189, People’s Republic of China

## Abstract

In the title compound, CH_3_NH_3_^+^·BF_4_^−^·C_12_H_24_O_6_, the methyl­ammonium cation makes three N—H⋯O hydrogen bonds to the 18-crown-6 mol­ecule. The –NH_3_^+^ and –CH_3_ groups of the cation adopt a staggered conformation. The F atoms of the tetra­fluoridoborate anion are disordered over two sets of sites in a 0.519 (11):0.481 (11) ratio. Weak C—H⋯F inter­actions occur in the crystal, which possibly correlate with the anion disorder.

## Related literature

For related structures, see: Henschel *et al.* (1999 ▶); Trueblood *et al.* (1982 ▶). For the possible relationship of the title compound to mol­ecular ferroelectrics, see: Wu *et al.* (2011 ▶).
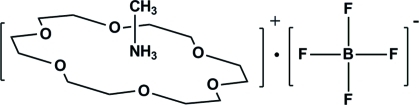


## Experimental

### 

#### Crystal data

CH_6_N^+^·BF_4_^−^·C_12_H_24_O_6_
*M* *_r_* = 383.19
Monoclinic, 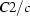

*a* = 24.375 (5) Å
*b* = 8.5404 (17) Å
*c* = 21.345 (4) Å
β = 116.90 (3)°
*V* = 3962.7 (14) Å^3^
*Z* = 8
Mo *K*α radiation
μ = 0.12 mm^−1^
*T* = 293 K
0.3 × 0.3 × 0.2 mm

#### Data collection

Rigaku Mercury CCD diffractometer
Absorption correction: multi-scan (*CrystalClear*; Rigaku, 2005 ▶) *T*_min_ = 0.489, *T*_max_ = 1.000
19676 measured reflections
4519 independent reflections
2155 reflections with *I* > 2σ(*I*)
*R*_int_ = 0.061

#### Refinement

*R*[*F* ^2^ > 2σ(*F* ^2^)] = 0.073
*wR*(*F* ^2^) = 0.226
*S* = 1.03
4519 reflections
265 parameters
H-atom parameters constrained
Δρ_max_ = 0.25 e Å^−3^
Δρ_min_ = −0.19 e Å^−3^

### 

Data collection: *CrystalClear* (Rigaku, 2005 ▶); cell refinement: *CrystalClear*; data reduction: *CrystalClear*; program(s) used to solve structure: *SHELXS97* (Sheldrick, 2008 ▶); program(s) used to refine structure: *SHELXL97* (Sheldrick, 2008 ▶); molecular graphics: *SHELXTL* (Sheldrick, 2008 ▶); software used to prepare material for publication: *SHELXL97*.

## Supplementary Material

Crystal structure: contains datablock(s) I, global. DOI: 10.1107/S1600536811054432/hb6552sup1.cif

Structure factors: contains datablock(s) I. DOI: 10.1107/S1600536811054432/hb6552Isup2.hkl

Additional supplementary materials:  crystallographic information; 3D view; checkCIF report

## Figures and Tables

**Table 1:** Hydrogen-bond geometry (Å, °)

*D*—H⋯*A*	*D*—H	H⋯*A*	*D*⋯*A*	*D*—H⋯*A*
N1—H1*C*⋯O2	0.89	1.98	2.867 (3)	171
N1—H1*B*⋯O6	0.89	1.99	2.866 (3)	168
N1—H1*A*⋯O4	0.89	1.99	2.876 (3)	171
C2—H2*B*⋯F2′^i^	0.97	2.41	3.345 (14)	163
C7—H7*B*⋯F3′	0.97	2.52	3.481 (13)	172
C9—H9*B*⋯F1′^ii^	0.97	2.49	3.306 (12)	142
C9—H9*B*⋯F2′^ii^	0.97	2.36	3.298 (17)	163
C11—H11*A*⋯F4′^iii^	0.97	2.47	3.393 (12)	158

Symmetry codes: (i) 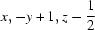; (ii) 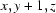; (iii) 
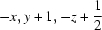
.

**Fig. 1.**
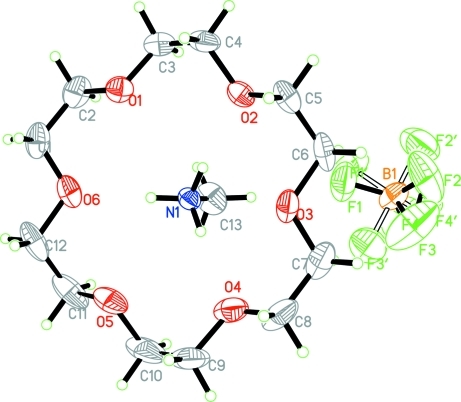
Molecular structure of the title compound with displacement ellipsoids drawn at the 30% probability level.

**Fig. 2.**
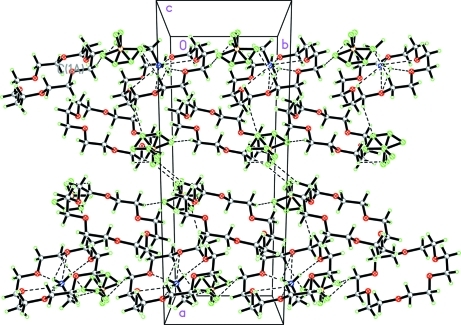
Crystal structure of the title compound with view along the c axis. Intermolecular interactions are shown as dashed lines.

## supplementary crystallographic information

### Comment

Molecular motion has proved to cause a rotation of the local structure to give rise to the formation of reversible structural phase transition from high-temperature disordered state to low temperature ordered state. Only a small part of compounds in which the components can be arranged in a disordered status at a relative high temperature and in an ordered one at a relative low temperature have been found until now. The transition from the disordered arrangement to the ordered one results in sharp change in the physical properties of compounds (e.g. Wu *et al.*, 2011). The protonated CH_3_—NH_3_^+^ cation can be easily anchored in the cavity of 18-crown-6, as a result of strong N—H···O hydrogen-bonding interactions. In the protonated CH_3_—NH_3_^+^, –NH_3_^+^ can be fixed firmly by 18-crown-6 which is an excellent molecular-based stator *via* N—H···O hydrogen-bonding interactions forming a stationary axis along which the rest of CH_3_—NH_3_^+^ cation can rotate freely. The introduction of a disordered group in the compounds results in the potential for the order-disorder transition due to the fact that the freezing of a disordered group at low temperature forces significant orientational motions of the group and thus may induce the formation of the ferroelectric phase. As part of our search for simple ferroelectric compounds we have investigated the title compound and reported its room temperature structure.

In this crystal structure, the nitrogen of the –NH_3_^+^ group lies 1.043 Å from the plane of the O atoms of the crown ring, in contrast to the analogous complex (Henschel *et al.*, 1999) and analogous perchlorate hemihytrate (Trueblood *et al.*, 1982), where the corresponding distance is 0.981 Å and 0.68 Å individually.

The anion and one cation are shown in Fig. 1 with the hydrogen bonds listed in Table 1. The existence of N—H···O hydrogen-bonding interactions and weak C—H···F interactions helps to make the substance more stable, and thus forms a three-dimensional structure. The components are held together by N—H···O hydrogen bonds and weak C—H···F interactions, forming a 1:1:1 aggregate. The aggregates are further connected by weak C—H···F interactions, and thus forms a complex spatial geometry.

### Experimental

(C_12_H_24_O_6_.CH_3_NH_3_^+^).(BF^-^_4_) was formed from a mixture of C_12_H_24_O_6_ (264.32 mg, 1.00 mmol), CH_3_NH_2_ (8 mL, 40% aqueous solution), tetrafluoridoborate (10 mL, 48% aqueous solution) and distilled water (5 ml), which was stirred a few minutes at room temperature, giving a clear transparent solution. After evaporation for a few days, block colorless crystals suitable for X-ray diffraction were obtained in about 60% yield and filtered and washed with distilled water.

### Refinement

H atoms bound to carbon and nitrogen were placed at idealized positions [C—H = 0.96–0.97 Å, N—H = 0.89 Å] and allowed to ride on their parent atoms with *U*_iso_ fixed at 1.2 *U*_eq_(C,*N*).

### Crystal data

**Table:**

CH_6_N^+^·BF_4_^−^·C_12_H_24_O_6_	*F*(000) = 1632
*M**_r_* = 383.19	*D*_x_ = 1.285 Mg m^−^^3^
Monoclinic, *C*2/*c*	Mo *K*α radiation, λ = 0.71073 Å
Hall symbol: -C 2yc	Cell parameters from 3450 reflections
*a* = 24.375 (5) Å	θ = 6.2–55.3°
*b* = 8.5404 (17) Å	µ = 0.12 mm^−^^1^
*c* = 21.345 (4) Å	*T* = 293 K
β = 116.90 (3)°	Block, colorless
*V* = 3962.7 (14) Å^3^	0.3 × 0.3 × 0.2 mm
*Z* = 8	

### Data collection

**Table:**

Rigaku Mercury CCD diffractometer	4519 independent reflections
Radiation source: fine-focus sealed tube	2155 reflections with *I* > 2σ(*I*)
graphite	*R*_int_ = 0.061
ω scans	θ_max_ = 27.5°, θ_min_ = 3.1°
Absorption correction: multi-scan (*CrystalClear*; Rigaku, 2005)	*h* = −31→31
*T*_min_ = 0.489, *T*_max_ = 1.000	*k* = −11→11
19676 measured reflections	*l* = −27→27

### Refinement

**Table:**

Refinement on *F*^2^	Primary atom site location: structure-invariant direct methods
Least-squares matrix: full	Secondary atom site location: difference Fourier map
*R*[*F*^2^ > 2σ(*F*^2^)] = 0.073	Hydrogen site location: inferred from neighbouring sites
*wR*(*F*^2^) = 0.226	H-atom parameters constrained
*S* = 1.03	*w* = 1/[σ^2^(*F*_o_^2^) + (0.0967*P*)^2^ + 1.8449*P*] where *P* = (*F*_o_^2^ + 2*F*_c_^2^)/3
4519 reflections	(Δ/σ)_max_ < 0.001
265 parameters	Δρ_max_ = 0.25 e Å^−^^3^
0 restraints	Δρ_min_ = −0.19 e Å^−^^3^

### Special details

**Table:**

Geometry. All e.s.d.'s (except the e.s.d. in the dihedral angle between two l.s. planes) are estimated using the full covariance matrix. The cell e.s.d.'s are taken into account individually in the estimation of e.s.d.'s in distances, angles and torsion angles; correlations between e.s.d.'s in cell parameters are only used when they are defined by crystal symmetry. An approximate (isotropic) treatment of cell e.s.d.'s is used for estimating e.s.d.'s involving l.s. planes.
Refinement. Refinement of *F*^2^ against ALL reflections. The weighted *R*-factor *wR* and goodness of fit *S* are based on *F*^2^, conventional *R*-factors *R* are based on *F*, with *F* set to zero for negative *F*^2^. The threshold expression of *F*^2^ > σ(*F*^2^) is used only for calculating *R*-factors(gt) *etc*. and is not relevant to the choice of reflections for refinement. *R*-factors based on *F*^2^ are statistically about twice as large as those based on *F*, and *R*- factors based on ALL data will be even larger.

### Fractional atomic coordinates and isotropic or equivalent isotropic displacement parameters (Å^2^)

**Table:**

	*x*	*y*	*z*	*U*_iso_*/*U*_eq_	Occ. (<1)
B1	0.0812 (2)	0.6121 (6)	0.4200 (2)	0.0824 (12)	
C1	0.09781 (16)	0.9413 (5)	0.04075 (16)	0.0815 (11)	
H1D	0.1361	0.9848	0.0451	0.098*	
H1E	0.0666	0.9547	−0.0074	0.098*	
C2	0.10575 (15)	0.7737 (4)	0.05902 (16)	0.0798 (10)	
H2A	0.0684	0.7324	0.0584	0.096*	
H2B	0.1138	0.7162	0.0249	0.096*	
C3	0.16487 (15)	0.5967 (4)	0.14898 (18)	0.0713 (9)	
H3A	0.1717	0.5339	0.1153	0.086*	
H3B	0.1285	0.5573	0.1513	0.086*	
C4	0.21812 (15)	0.5856 (3)	0.21816 (19)	0.0710 (9)	
H4A	0.2285	0.4764	0.2302	0.085*	
H4B	0.2532	0.6367	0.2171	0.085*	
C5	0.25467 (14)	0.6571 (4)	0.33741 (17)	0.0726 (9)	
H5A	0.2888	0.7145	0.3369	0.087*	
H5B	0.2679	0.5500	0.3513	0.087*	
C6	0.23720 (16)	0.7287 (4)	0.38822 (16)	0.0786 (11)	
H6A	0.2014	0.6757	0.3867	0.094*	
H6B	0.2707	0.7183	0.4353	0.094*	
C7	0.2087 (2)	0.9725 (5)	0.41965 (19)	0.0952 (12)	
H7A	0.2418	0.9602	0.4668	0.114*	
H7B	0.1715	0.9291	0.4185	0.114*	
C8	0.1995 (2)	1.1415 (5)	0.4007 (2)	0.1035 (14)	
H8A	0.1915	1.1987	0.4349	0.124*	
H8B	0.2364	1.1843	0.4007	0.124*	
C9	0.1367 (3)	1.3161 (4)	0.3089 (3)	0.1140 (16)	
H9A	0.1722	1.3590	0.3058	0.137*	
H9B	0.1294	1.3782	0.3424	0.137*	
C10	0.0821 (3)	1.3234 (5)	0.2390 (3)	0.1215 (18)	
H10A	0.0477	1.2691	0.2405	0.146*	
H10B	0.0702	1.4316	0.2261	0.146*	
C11	0.04701 (16)	1.2532 (5)	0.1191 (3)	0.1032 (15)	
H11A	0.0336	1.3600	0.1049	0.124*	
H11B	0.0126	1.1944	0.1183	0.124*	
C12	0.06760 (16)	1.1822 (4)	0.0705 (2)	0.0937 (13)	
H12A	0.0360	1.1944	0.0224	0.112*	
H12B	0.1045	1.2344	0.0749	0.112*	
C13	0.06353 (16)	0.8461 (5)	0.2238 (2)	0.0916 (12)	
H13A	0.0719	0.7970	0.2677	0.137*	
H13B	0.0485	0.7693	0.1870	0.137*	
H13C	0.0331	0.9264	0.2137	0.137*	
F1	0.0811 (5)	0.6402 (13)	0.3601 (5)	0.131 (3)	0.481 (11)
F2	0.1331 (3)	0.5661 (14)	0.4620 (5)	0.165 (5)	0.481 (11)
F3	0.1255 (7)	0.705 (3)	0.4670 (9)	0.198 (8)	0.481 (11)
F4	0.0293 (5)	0.6160 (16)	0.4217 (8)	0.156 (5)	0.481 (11)
F1'	0.0550 (4)	0.5665 (13)	0.3505 (4)	0.128 (3)	0.519 (11)
F2'	0.1037 (8)	0.4578 (13)	0.4316 (7)	0.245 (7)	0.519 (11)
F3'	0.0814 (5)	0.7788 (7)	0.4154 (6)	0.131 (3)	0.519 (11)
F4'	0.0385 (5)	0.5822 (11)	0.4440 (7)	0.134 (4)	0.519 (11)
N1	0.12000 (9)	0.9158 (2)	0.22837 (11)	0.0495 (6)	
H1A	0.1325	0.9917	0.2604	0.074*	
H1B	0.1128	0.9553	0.1868	0.074*	
H1C	0.1491	0.8427	0.2408	0.074*	
O1	0.15612 (9)	0.7548 (2)	0.12755 (10)	0.0627 (6)	
O2	0.20466 (8)	0.6585 (2)	0.27007 (10)	0.0589 (5)	
O3	0.22339 (9)	0.8921 (2)	0.37129 (10)	0.0678 (6)	
O4	0.14840 (12)	1.1580 (3)	0.33206 (15)	0.0861 (8)	
O5	0.09714 (10)	1.2516 (2)	0.18839 (14)	0.0836 (7)	
O6	0.07986 (9)	1.0197 (3)	0.08667 (11)	0.0745 (6)	

### Atomic displacement parameters (Å^2^)

**Table:**

	*U*^11^	*U*^22^	*U*^33^	*U*^12^	*U*^13^	*U*^23^
B1	0.093 (4)	0.091 (4)	0.079 (3)	−0.017 (3)	0.052 (3)	−0.021 (3)
C1	0.077 (2)	0.108 (3)	0.0402 (17)	−0.023 (2)	0.0092 (16)	0.0027 (17)
C2	0.072 (2)	0.099 (3)	0.0504 (18)	−0.0180 (19)	0.0124 (17)	−0.0165 (18)
C3	0.072 (2)	0.0590 (19)	0.086 (2)	0.0001 (16)	0.0379 (19)	−0.0157 (17)
C4	0.074 (2)	0.0512 (17)	0.102 (3)	0.0160 (15)	0.052 (2)	−0.0005 (17)
C5	0.0608 (19)	0.0652 (19)	0.072 (2)	0.0068 (15)	0.0130 (17)	0.0232 (16)
C6	0.080 (2)	0.079 (2)	0.0510 (18)	−0.0188 (18)	0.0075 (17)	0.0282 (17)
C7	0.113 (3)	0.119 (3)	0.062 (2)	−0.033 (3)	0.047 (2)	−0.015 (2)
C8	0.140 (4)	0.108 (3)	0.087 (3)	−0.042 (3)	0.072 (3)	−0.049 (2)
C9	0.170 (5)	0.060 (2)	0.163 (5)	0.000 (3)	0.119 (4)	−0.030 (3)
C10	0.143 (4)	0.060 (2)	0.209 (6)	0.039 (3)	0.121 (5)	0.017 (3)
C11	0.053 (2)	0.070 (2)	0.160 (4)	0.0206 (18)	0.025 (3)	0.047 (3)
C12	0.0542 (19)	0.087 (3)	0.100 (3)	0.0011 (19)	0.000 (2)	0.055 (2)
C13	0.071 (2)	0.091 (3)	0.121 (3)	−0.0077 (19)	0.051 (2)	0.014 (2)
F1	0.164 (8)	0.154 (8)	0.084 (5)	−0.001 (5)	0.064 (6)	0.024 (5)
F2	0.077 (4)	0.167 (9)	0.153 (7)	−0.012 (5)	−0.035 (4)	0.070 (6)
F3	0.143 (9)	0.293 (16)	0.188 (12)	−0.136 (11)	0.102 (9)	−0.138 (13)
F4	0.087 (5)	0.236 (12)	0.166 (9)	−0.025 (5)	0.076 (5)	−0.021 (7)
F1'	0.125 (6)	0.157 (8)	0.095 (4)	0.004 (4)	0.044 (4)	−0.051 (5)
F2'	0.445 (18)	0.148 (8)	0.296 (12)	0.147 (10)	0.302 (14)	0.114 (8)
F3'	0.156 (7)	0.091 (4)	0.154 (6)	−0.039 (4)	0.077 (6)	−0.006 (4)
F4'	0.207 (10)	0.103 (4)	0.173 (8)	−0.099 (6)	0.156 (8)	−0.088 (5)
N1	0.0497 (12)	0.0450 (12)	0.0531 (13)	0.0062 (10)	0.0227 (11)	0.0051 (10)
O1	0.0595 (12)	0.0602 (13)	0.0624 (13)	−0.0057 (9)	0.0223 (10)	−0.0088 (10)
O2	0.0451 (10)	0.0542 (11)	0.0685 (13)	0.0106 (8)	0.0178 (10)	0.0096 (9)
O3	0.0808 (14)	0.0767 (14)	0.0451 (11)	−0.0172 (11)	0.0277 (10)	0.0049 (10)
O4	0.115 (2)	0.0612 (14)	0.110 (2)	−0.0184 (13)	0.0749 (18)	−0.0261 (13)
O5	0.0680 (14)	0.0553 (13)	0.130 (2)	0.0189 (11)	0.0476 (15)	0.0133 (13)
O6	0.0686 (14)	0.0717 (14)	0.0649 (13)	−0.0036 (11)	0.0140 (11)	0.0222 (11)

### Geometric parameters (Å, °)

**Table:**

B1—F2	1.238 (8)	C7—O3	1.414 (4)
B1—F4	1.281 (11)	C7—C8	1.489 (6)
B1—F1	1.299 (9)	C7—H7A	0.9700
B1—F3	1.348 (8)	C7—H7B	0.9700
B1—F4'	1.376 (10)	C8—O4	1.437 (5)
B1—F1'	1.380 (8)	C8—H8A	0.9700
B1—F2'	1.406 (9)	C8—H8B	0.9700
B1—F3'	1.428 (7)	C9—O4	1.422 (5)
C1—O6	1.410 (4)	C9—C10	1.486 (7)
C1—C2	1.474 (5)	C9—H9A	0.9700
C1—H1D	0.9700	C9—H9B	0.9700
C1—H1E	0.9700	C10—O5	1.427 (5)
C2—O1	1.431 (4)	C10—H10A	0.9700
C2—H2A	0.9700	C10—H10B	0.9700
C2—H2B	0.9700	C11—O5	1.430 (5)
C3—O1	1.411 (3)	C11—C12	1.473 (6)
C3—C4	1.463 (5)	C11—H11A	0.9700
C3—H3A	0.9700	C11—H11B	0.9700
C3—H3B	0.9700	C12—O6	1.429 (4)
C4—O2	1.432 (3)	C12—H12A	0.9700
C4—H4A	0.9700	C12—H12B	0.9700
C4—H4B	0.9700	C13—N1	1.461 (4)
C5—O2	1.402 (3)	C13—H13A	0.9600
C5—C6	1.466 (5)	C13—H13B	0.9600
C5—H5A	0.9700	C13—H13C	0.9600
C5—H5B	0.9700	N1—H1A	0.8900
C6—O3	1.443 (4)	N1—H1B	0.8900
C6—H6A	0.9700	N1—H1C	0.8900
C6—H6B	0.9700		
			
F2—B1—F4	133.3 (10)	O3—C6—H6A	109.8
F2—B1—F1	108.5 (8)	C5—C6—H6A	109.8
F4—B1—F1	117.4 (9)	O3—C6—H6B	109.8
F2—B1—F3	55.5 (12)	C5—C6—H6B	109.8
F4—B1—F3	115.6 (8)	H6A—C6—H6B	108.3
F1—B1—F3	105.5 (7)	O3—C7—C8	109.2 (3)
F2—B1—F4'	111.6 (8)	O3—C7—H7A	109.8
F4—B1—F4'	21.9 (12)	C8—C7—H7A	109.8
F1—B1—F4'	137.4 (8)	O3—C7—H7B	109.8
F3—B1—F4'	108.6 (6)	C8—C7—H7B	109.8
F2—B1—F1'	120.9 (8)	H7A—C7—H7B	108.3
F4—B1—F1'	93.2 (8)	O4—C8—C7	109.0 (3)
F1—B1—F1'	36.9 (4)	O4—C8—H8A	109.9
F3—B1—F1'	142.3 (7)	C7—C8—H8A	109.9
F4'—B1—F1'	106.7 (6)	O4—C8—H8B	109.9
F2—B1—F2'	52.0 (7)	C7—C8—H8B	109.9
F4—B1—F2'	109.4 (10)	H8A—C8—H8B	108.3
F1—B1—F2'	100.0 (6)	O4—C9—C10	109.7 (4)
F3—B1—F2'	107.4 (16)	O4—C9—H9A	109.7
F4'—B1—F2'	93.6 (8)	C10—C9—H9A	109.7
F1'—B1—F2'	83.0 (9)	O4—C9—H9B	109.7
F2—B1—F3'	109.4 (8)	C10—C9—H9B	109.7
F4—B1—F3'	90.6 (7)	H9A—C9—H9B	108.2
F1—B1—F3'	75.4 (6)	O5—C10—C9	108.8 (3)
F3—B1—F3'	55.9 (9)	O5—C10—H10A	109.9
F4'—B1—F3'	103.7 (6)	C9—C10—H10A	109.9
F1'—B1—F3'	102.9 (6)	O5—C10—H10B	109.9
F2'—B1—F3'	159.0 (10)	C9—C10—H10B	109.9
O6—C1—C2	108.8 (3)	H10A—C10—H10B	108.3
O6—C1—H1D	109.9	O5—C11—C12	108.7 (3)
C2—C1—H1D	109.9	O5—C11—H11A	109.9
O6—C1—H1E	109.9	C12—C11—H11A	109.9
C2—C1—H1E	109.9	O5—C11—H11B	109.9
H1D—C1—H1E	108.3	C12—C11—H11B	109.9
O1—C2—C1	109.2 (2)	H11A—C11—H11B	108.3
O1—C2—H2A	109.8	O6—C12—C11	109.2 (3)
C1—C2—H2A	109.8	O6—C12—H12A	109.8
O1—C2—H2B	109.8	C11—C12—H12A	109.8
C1—C2—H2B	109.8	O6—C12—H12B	109.8
H2A—C2—H2B	108.3	C11—C12—H12B	109.8
O1—C3—C4	108.9 (2)	H12A—C12—H12B	108.3
O1—C3—H3A	109.9	N1—C13—H13A	109.5
C4—C3—H3A	109.9	N1—C13—H13B	109.5
O1—C3—H3B	109.9	H13A—C13—H13B	109.5
C4—C3—H3B	109.9	N1—C13—H13C	109.5
H3A—C3—H3B	108.3	H13A—C13—H13C	109.5
O2—C4—C3	110.1 (2)	H13B—C13—H13C	109.5
O2—C4—H4A	109.6	C13—N1—H1A	109.5
C3—C4—H4A	109.6	C13—N1—H1B	109.5
O2—C4—H4B	109.6	H1A—N1—H1B	109.5
C3—C4—H4B	109.6	C13—N1—H1C	109.5
H4A—C4—H4B	108.2	H1A—N1—H1C	109.5
O2—C5—C6	110.2 (3)	H1B—N1—H1C	109.5
O2—C5—H5A	109.6	C3—O1—C2	111.9 (2)
C6—C5—H5A	109.6	C5—O2—C4	113.1 (2)
O2—C5—H5B	109.6	C7—O3—C6	113.2 (3)
C6—C5—H5B	109.6	C9—O4—C8	113.1 (3)
H5A—C5—H5B	108.1	C10—O5—C11	112.7 (3)
O3—C6—C5	109.3 (2)	C1—O6—C12	112.9 (3)

### Hydrogen-bond geometry (Å, °)

**Table:**

*D*—H···*A*	*D*—H	H···*A*	*D*···*A*	*D*—H···*A*
N1—H1C···O2	0.89	1.98	2.867 (3)	171
N1—H1B···O6	0.89	1.99	2.866 (3)	168
N1—H1A···O4	0.89	1.99	2.876 (3)	171
C2—H2B···F2'^i^	0.97	2.41	3.345 (14)	163
C7—H7B···F3'	0.97	2.52	3.481 (13)	172
C9—H9B···F1'^ii^	0.97	2.49	3.306 (12)	142
C9—H9B···F2'^ii^	0.97	2.36	3.298 (17)	163
C11—H11A···F4'^iii^	0.97	2.47	3.393 (12)	158

Symmetry codes: (i) *x*, −*y*+1, *z*−1/2; (ii) *x*, *y*+1, *z*; (iii) −*x*, *y*+1, −*z*+1/2.

## References

[bb1] Henschel, D., Wijaya, K., Jones, P. G. & Blaschette, A. (1999). *Acta Cryst.* C**55**, 664–668.

[bb2] Rigaku (2005). *CrystalClear* Rigaku Corporation, Tokyo, Japan.

[bb3] Sheldrick, G. M. (2008). *Acta Cryst.* A**64**, 112–122. 10.1107/S0108767307043930 18156677

[bb4] Trueblood, K.-N., Knobler, C.-B., Lawrence, D.-S. & Stevens, R.-V. (1982). *J. Am. Chem. Soc.* **104**, 1355–1362.

[bb5] Wu, D.-H., Ge, J.-Z., Cai, H.-L., Zhang, W. & Xiong, R.-G. (2011). *CrystEngComm*, **13**, 319–324.

